# Supplementary material for: Detailed characterization of the solution kinetics and thermodynamics of biotin, biocytin and HABA binding to avidin and streptavidin
Source: PLoS One. 2019 Feb 28;14(2):e0204194. doi: 10.1371/journal.pone.0204194 (PMC6394990; doi:10.1371/journal.pone.0204194)
Supplement: S1 Fig — The absorbance spectra of the unbound BFl, BcO and B7-DNAds*Fl are shown in dark blue and the respective bound complexes formed with SAV and AV in pink. The spectra are normalized to the calculated molar absorptivies. The distortion of the SAV-BFl absorbance spectrum (B panel) is caused by the presence of Fl2- and Fl1-. The labeled probes and protein concentrations were 1 μM and 10 μM, respectively. (DOCX) [file pone.0204194.s001.docx]

**Supporting Information S1 Fig.**

**S1 Fig. Absorbance spectra of dye-labeled B_7_ probes and respective complexes with AV and SAV.** The absorbance spectra of the unbound BFl, BcO and B_7_-DNA_ds_*Fl are shown in dark blue and the respective bound complexes formed with SAV and AV in pink. The spectra are normalized to the calculated molar absorptivies. The distortion of the SAV-BFl absorbance spectrum (B panel) is caused by the presence of Fl^2-^ and Fl^1-^. The labeled probes and protein concentrations were 1 µM and 10 µM, respectively.
