# Supplementary material for: Detailed characterization of the solution kinetics and thermodynamics of biotin, biocytin and HABA binding to avidin and streptavidin
Source: PLoS One. 2019 Feb 28;14(2):e0204194. doi: 10.1371/journal.pone.0204194 (PMC6394990; doi:10.1371/journal.pone.0204194)
Supplement: S2 Fig — The normalized spectra of the unbound BFl, BcO and B7-DNAds*Fl are shown in blue and their respective bound complexes formed with SAV and AV in pink. The probe and protein concentrations were 20 nM and 1040 nM (AB1 filling model), respectively. (DOCX) [file pone.0204194.s002.docx]

**Supporting Information S2 Fig.**

**S2 Fig. Fluorescence emission spectra of dye-labeled B_7_ and respective complexes with AV and SAV.** The normalized spectra of the unbound BFl, BcO and B_7_-DNA_ds_*Fl are shown in blue and their respective bound complexes formed with SAV and AV in pink. The probe and protein concentrations were 20 nM and 1040 nM (AB_1_ filling model), respectively.
