# Supplementary material for: Detailed characterization of the solution kinetics and thermodynamics of biotin, biocytin and HABA binding to avidin and streptavidin
Source: PLoS One. 2019 Feb 28;14(2):e0204194. doi: 10.1371/journal.pone.0204194 (PMC6394990; doi:10.1371/journal.pone.0204194)
Supplement: S3 Fig — The dissociation reactions of AV complexes were carried out with a preformed complex of 20 nM BFl or BcO and 260 nM AV for a filling model of AB1 and challenged with unlabeled B7 at 2,000 nM. The koffdisplacement could not be detected and the corresponding koffreplacement (9 x 10−8 s-1) found by Green N. [35] is too slow to be determined by the our fluorescence anisotropy methodology. (DOCX) [file pone.0204194.s003.docx]

**Supporting Information S3 Fig.**

**S3 Fig. Dissociation reactions of AV-BcO and AV-BFl complexes by unlabeled B_7_ at 20 ºC.** The dissociation reactions of AV complexes were carried out with a preformed complex of 20 nM BFl or BcO and 260 nM AV for a filling model of AB_1_ and challenged with unlabeled B_7_ at 2,000 nM. The $k_{off}^{displacement}$ could not be detected and the corresponding $k_{off}^{replacement}$ (9 x 10^-8^ s^-1^) found by Green N. (The use of [14C]biotin for kinetic studies and for assay. Biochem J. 1963;89:585–91) was too slow to be determined by our fluorescence anisotropy methodology.
