# Supplementary material for: Detailed characterization of the solution kinetics and thermodynamics of biotin, biocytin and HABA binding to avidin and streptavidin
Source: PLoS One. 2019 Feb 28;14(2):e0204194. doi: 10.1371/journal.pone.0204194 (PMC6394990; doi:10.1371/journal.pone.0204194)
Supplement: S1 Table — The fluorescence lifetimes are shown in nanoseconds and were obtained in solution. (DOCX) [file pone.0204194.s004.docx]

**Supporting Information S1 Table.**

**S1. Table. Lifetimes of dye-labeled biotin probes and protein complexes**

| **Sample** | **α_1_** | **τ_1_ (ns)** | **α_2_** | **τ_2_ (ns)** | **Σα_i_τ_i_  *^a^* (ns)** |
| --- | --- | --- | --- | --- | --- |
| **SAV-BFl** | 0.61 ± 0.03 | 4.10 ± 0.02 | 0.39 ± 0.03 | 3.1 ± 0.02 | 3.70 ± 0.05 |
| **B_7_-DNA_ss_*Fl-3’** *^b^* | 1.0 | 3.99 ± 0.03 | NA | NA | 3.99 ± 0.03 |
| **AV- B_7_-DNA_ss_*Fl-3’** *^c^* | 0.22 ± 0.01 | 1.12 ± 0.19 | 0.78 ± 0.01 | 4.15 ± 0.03 | 3.48 ± 0.06 |
| **SAV- B_7_-DNA_ss_*Fl-3’** | 0.50 ± 0.01 | 0.72 ± 0.01 | 0.50 ± 0.01 | 3.78 ± 0.01 | 2.25 ± 0.04 |
| **B_7_-DNA_ds_*Fl-3’** | 0.20 ± 0.02 | 1.10 ± 0.05 | 0.80 ± 0.02 | 3.62 ± 0.01 | 3.12 ± 0.08 |
| **AV- B_7_-DNA_ds_*Fl-3’** | 0.09 ± 0.01 | 1.27 ± 0.11 | 0.91 ± 0.01 | 4.05 ± 0.01 | 3.80 ± 0.05 |
| **SAV- B_7_-DNA_ds_*Fl-3’** | 0.12 ± 0.01 | 2.29 ± 0.02 | 0.88 ± 0.01 | 4.08 ± 0.01 | 3.86 ± 0.01 |

The fluorescence lifetimes in nanoseconds (ns) were acquired in solution at nanomolar concentrations and they were temperature independent from 10 °C to 25 °C

*^a^* Σα_i_τ_i_, is the integrated lifetime.

*^b^* ss, single strand of the 14mer*Fl.

*^c^* ds, duplex strand of the 14mer*Fl.
